# RNA Editing in Cancer Progression

**DOI:** 10.3390/cancers15215277

**Published:** 2023-11-03

**Authors:** Valentina Frezza, Lidia Chellini, Arianna Del Verme, Maria Paola Paronetto

**Affiliations:** 1 Laboratory of Molecular and Cellular Neurobiology, Fondazione Santa Lucia, CERC, Via del Fosso di Fiorano, 64, 00143 Rome, Italy; valentinafrezza.83@gmail.com (V.F.); chellinilidia@gmail.com (L.C.); ariannadelverme02@gmail.com (A.D.V.); 2 Department of Movement, Human and Health Sciences, University of Rome “Foro Italico”, Piazza Lauro de Bosis, 15, 00135 Rome, Italy

**Keywords:** RNA editing, splicing, ADAR, DHX9, cancer

## Abstract

**Simple Summary:**

Recent evidence from whole-exome and whole-genome sequencing approaches revealed a complex genomic landscape for many cancers. In addition to somatic mutations and alternative splicing changes, genetic information can also be altered by RNA editing, which enables alterations of genome information in a very dynamic and flexible way. Influenced by both external factors and microenvironmental signals, RNA editing deeply contributes to cancer morphological plasticity.

**Abstract:**

Coding and noncoding RNA molecules play their roles in ensuring cell function and tissue homeostasis in an ordered and systematic fashion. RNA chemical modifications can occur both at bases and ribose sugar, and, similarly to DNA and histone modifications, can be written, erased, and recognized by the corresponding enzymes, thus modulating RNA activities and fine-tuning gene expression programs. RNA editing is one of the most prevalent and abundant forms of post-transcriptional RNA modification in normal physiological processes. By altering the sequences of mRNAs, it makes them different from the corresponding genomic template. Hence, edited mRNAs can produce protein isoforms that are functionally different from the corresponding genome-encoded variants. Abnormalities in regulatory enzymes and changes in RNA-modification patterns are closely associated with the occurrence and development of various human diseases, including cancer. To date, the roles played by RNA modifications in cancer are gathering increasing interest. In this review, we focus on the role of RNA editing in cancer transformation and provide a new perspective on its impact on tumorigenesis, by regulating cell proliferation, differentiation, invasion, migration, stemness, metabolism, and drug resistance.

## 1. Introduction

The genome-wide complexity is greatly expanded by RNA processing features, including RNA editing. The output information deriving from a single gene depends on how the pre-mRNA is processed, which is strictly connected to the specific cell type, location, and fate, indicating that the cellular environment determines in time and space which splice sites are recognized and which codons are edited [1]. In this context, each specific phenotype can be determined by processing features, in addition to genomic features. Defective genomic sequences can even be corrected into functional products by compensatory processing modifications, as shown for the mitochondrial genome of trypanosomes, whose transcripts, which contain frameshifts and stop codons, would be incapable of coding for active protein without extensive editing [2]. Hence, RNA editing deeply affects gene expression, and alterations in this process can be responsible for the activation of oncogenes and/or inactivation of tumor suppressors, thus contributing to cancer. In this review, we will examine the main RNA editing features involved in tumor progression, their impact on splicing choices, and how the management of these characteristics could be exploited for therapeutic proposal.

## 2. An Overview of RNA Editing

RNA editing comprises different types of irreversible post-transcriptional processing events that modify the RNA sequences and introduce mismatches, thus modifying the coding capacity of a given transcript. The term ‘RNA editing’ was first referred to as the insertion and deletion of uridine nucleotides in the trypanosome mitochondrial mRNA encoding the cytochrome oxidase subunit II (*coxII*) [3]. Later on, the insertion and deletion of uridines turned out to be a common mechanism of the mitochondrial RNA of kinetoplastid, required for introducing stop codons or new AUG initiation codons in the transcripts [4,5], thus leading to novel protein isoforms [6]. This process was initially thought to rely on the hybridization of a guide RNA with the pre-mRNA [7]; but, later on, the mechanism accounted for the enzymatic activity of the RNA-editing core complex (RECC) [8].

The A-to-I transition, which is the most common editing feature across Metazoa, was the last editing process identified [9]. This conversion is read by the translation machinery as a guanosine [9]. The phenomenon was initially described as a dsRNA unwinding or helicase activity. Thereafter, it was elucidated that the conversion was caused by a hydrolytic C6 deamination of adenosine to inosine in the nucleus [10], attributed to the enzymatic activity of double-stranded RNA-specific adenosine deaminases [10], collectively named adenosine deaminase that act on RNA (ADAR) [11]. In humans, three members of the ADAR family have been identified, and two other adenosine deaminase domain-containing (ADAD) proteins (ADAD1 and ADAD2) [12]. ADAR1 and ADAR2 are catalytically active, whereas ADAR3, ADAD1, and ADAD2 are not enzymatically active. ADAR1 and ADAR2 are almost ubiquitously expressed, whereas ADAR3 is expressed exclusively in the brain. Both ADAR1 and ADAR2 form active homodimers and comprise a repeated double-stranded RNA binding motif and a C-terminal catalytic domain [13]. Full-length ADAR1p150 and the shorter ADARp110 share an identical sequence. While a Zα domain is unique to ADAR1p150 and may affect the binding preference, both isoforms contain a Zβ domain, a Z-DNA–RNA binding domain containing the nuclear localization signal (NLS) [13,14,15,16]. In human cells, ADAR1 and ADAR2 are sequestered within the nucleolus, where they presumably bind dsRNA deriving from the association of small nucleolar RNAs (snoRNAs) and ribosomal RNA. As needed, they can move to the nucleoplasm near sites of active transcription where they target pre-mRNA substrates, thereby influencing their splicing and coding potential [17,18]. ADAR1 displays a slight preference for deaminating adenosines with 5′ neighboring A, U, or C and deaminates short RNAs more selectively than long RNAs, [19,20], whereas ADAR2 favors adenosines with 3′ neighboring U and G [13].

There are two main forms of A-to-I editing: site selective and hyperediting. The site-selective editing targets determined adenosines within a short region of the transcript, without other neighboring deaminated adenosines nearby. This type of editing is less common and occurs in conserved coding regions displaying adjacent stem-loop structures shaped by bulges and mismatches [21,22]. Conversely, hyperediting is the most represented editing feature and produces enrichments of edited adenosines in extended regions (usually 10 or more edited adenosines in 100 bp) [23,24]. Hyperediting occurs within introns and untranslated regions (UTRs) [25,26,27]. Notably, upstream of genes and in intronic regions, inverted *Alu* repeats represent privileged sites for editing, since they tend to pair and form double-strand structures [28].

In addition to uridine insertion/deletion, a cytosine to uridine transition (C to U) was identified in the mRNA encoding the intestinal Apolipoprotein B-48 (APOB), leading to a premature termination codon [29,30]. APOB is a component of the plasma lipoproteins essential for the transport of cholesterol and triglycerides [30]. There are two main forms of APOB: APOB100 and the shorter APOB48, resulting from the deamination of C to U in the APOB mRNA, which causes the change of a glutamine residue into a stop codon [31]. Interestingly, in humans, this editing event acquires tissue-specific characteristics, since it occurs in the small intestine but not in the liver [32], where the APOB100 isoform is used to assemble the very-low-density lipoprotein (VLDL) that is necessary for the transport of endogenously synthesized triglycerides and cholesterol. In wheat mitochondrial mRNAs, the C-to-U transition causes changes in CGG into UGG codons, thus encoding tryptophan instead of arginine. The C-to-U substitution in the APOB mRNA was initially explained as a sequence-specific cytidine deamination [33]. Additional studies demonstrated that it was based on an enzymatic activity requiring zinc ions, not RNA guide cofactor, and having a 27 kDa cytidine deaminase subunit, conserved in bacteriophage, bacteria, yeast, and mammals [32,34]. This catalytic component of the editing enzyme was named Apobec-1 (apolipoprotein B mRNA-editing catalytic polypeptide-1) [35], and requires the auxiliary protein ACF for the docking of its target cytidines. Together, Apobec-1 and ACF form the minimal editosome functional assembly in vitro [36]. In humans, the APOBEC family of cytidine deaminases displays a tissue-specific expression pattern [37].

RNA editing plays a key role in generating molecular complexity in eukaryote transcriptomes. In a cell, not all adenosines at a given site are edited in every transcript, thus leading to a mixture of cellular transcripts. Rather than a static control of gene expression, A-to-I editing allows for dynamically rewiring the genetic code in a cell-type-specific manner. During normal development, and in both neurological diseases and cancers, the extent of RNA editing does not directly correlate with levels of the substrate mRNA or the editing enzymes, but cellular factors are required for the spatiotemporal regulation of editing efficiency. Recent studies have suggested that *cis*- and *trans*-acting RNA elements, as well as RNA-binding proteins (RBPs), can modulate RNA-editing efficiency in vivo. In addition, duplexed inverted *Alu* repeats act as ADAR recruitment elements, enhancing editing efficiency at adjacent sites [38,39]. 

Remarkably, the low basic level of editing detected throughout the transcriptome is a source for adaptive evolution, where cellular factors and hairpin structures made by *Alu* elements can shape. Unlike genetic mutations, the genetic variation introduced through editing occurs at a low evolutionary cost since production of the wild-type protein is retained and both isoforms (edited and nonedited) exist and may play a role in shaping the transcriptomic landscape.

## 3. Dysregulation of RNA Editing in Cancer Progression

**Figure 1:**
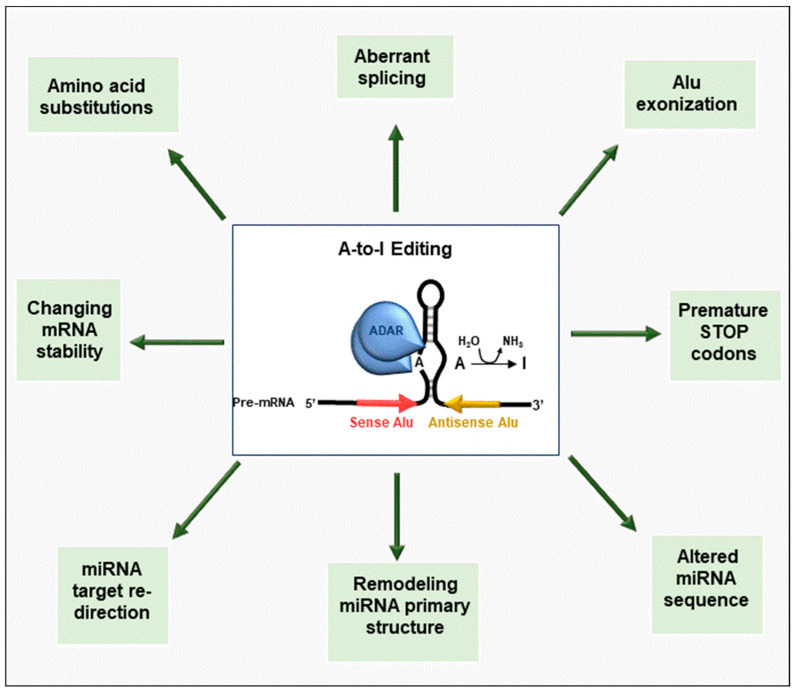
Graphical summary of RNA-editing mechanisms involved in cancer initiation and progression. Aberrant editing produces RNA modifications perturbing a wide range of biological processes involved in tumor behavior. Since edited adenosine is recognized as guanosine during translation, A-to-I RNA editing can contribute to the diversification of the transcriptome, thus implementing protein diversity. In particular, A-to-I RNA editing can not only alter an open reading frame or introduce premature stop codons but also create aberrant splicing isoforms or introduce novel exons derived from *Alu*-containing introns. A-to-I RNA editing can also influence mRNA expression levels, affecting mRNA stability. A-to-I RNA editing controls gene expression by modifying miRNAs, affecting their primary structure and processing, or by causing target redirection, which then leads to altered expression of their target mRNAs. References for each physiological function are indicated.

Deregulation of the RNA-editing process plays a pivotal role in cancer pathogenesis (Figure 1). An alteration in RNA sequences induced by the editing machinery profoundly affects gene expression, contributing to the acquisition of a tumorigenic and aggressive phenotype. In this regard, RNA editing can affect transcript stability and produce changes in the amino acid sequence of the translated protein. Furthermore, a dysregulated RNA-editing pattern can affect splice-site recognition and microRNA (miRNA) targets or even miRNA seed sequences [40].

The expression of the adenosine-to-inosine (A-to-I) RNA-editing regulator ADAR was found altered in different types of cancer [40,41,42,43,44]. To test its oncogenic properties, the overexpression of ADAR1 isoforms was performed in mice and did not result in cancer initiation [45], supporting the hypothesis that increased ADAR1 expression could be a consequence of tumor formation. In nonsmall-cell lung cancer (NSCLC), the deaminase activity of ADAR on an intronic site is critical for the stabilization and increase of focal adhesion kinase (FAK) transcript [46]. Activation of FAK signaling promotes lung cancer cell invasiveness. 

A-to-I editing in the *SLC22A3* gene operated by ADAR2 was found associated with reduced RNA transcription in familial esophageal squamous cell carcinoma (ESCC) [47]. Since SLC22A3 acts as a metastasis suppressor, a decrease in its expression facilitates cell invasion and metastasis formation [47]. ADAR1 overexpression correlated with increased editing was also described in breast cancer cells [48]. In particular, editing by ADAR1 was enhanced in the 3′UTR of cancer-related transcripts, including *ATM*, *GINS4*, and *POLH*. The 3′UTRs regulatory regions play crucial roles in mRNA stability and translation efficiency and are responsible for the recruitment of different protein complexes onto the mRNA [48]. A-to-I editing of the 3′UTR of *ATM* and *POLH* transcripts was able to modulate their expression rate, whereas editing of *GINS4* was associated with a decrease in mRNA stability [48]. Whether or not this was due to changes in the binding of specific RNA binding proteins (RBPs) has not been unraveled yet. In addition, in astrocytoma cells, ADAR2 promotes *CDC14B* editing with a concomitant increase in its expression [49]. The phosphatase CDC14B leads to Skp2 degradation and p21/p27 upregulation, indicating the essential role of ADAR2 in the inhibition of tumor growth [49].

In A-to-I editing, the edited inosine is recognized as guanosine during the translation, resulting in amino acid substitution and conferring to the RNA-editing process the ability to produce protein isoform variability. In glioblastoma multiforme (GBM), which is the most aggressive form of astrocytoma; the glutamate receptor subunit B (GluR-B) transcript was found underedited at the Q/R site [50]. The activity of ADAR2 modifies the CAG codon encoding glutamine (Q) into CIG, encoding arginine (R). Hence, the decreased editing in this position can cause alteration in Ca^2+^ permeability and affect the activity of the receptor, as observed in tumor-derived tissues compared to a control [50]. The level of RNA editing in the GluR-B and *GluR-6* transcripts was also analyzed in pediatric astrocytoma [44], where decreased ADAR2 activity was associated with underedited *GluR-B* and *GluR-6*, compared to control tissues. Loss of editing in the *GluR-B* transcript was sufficient to increase astrocytoma invasiveness in vivo [51]. Although overexpression of ADAR2 consistently decreased the cell growth rate of astrocytoma cell lines [44], astrocytoma patients did not show changes in ADAR2 expression, but only its activity was impaired. On the other hand, a significant increase in ADAR1 and ADAR3 was found in these patients, which positively correlated with the severity of the tumors. Thus, a high level of ADAR1 in astrocytoma could affect the specific editing activity of ADAR2.

Remarkably, in astrocytoma, the human bladder cancer-associated protein (BLCAP) transcript showed decreased editing [52]. These editing events, dependent on ADAR1 and ADAR2 activities, were found in both coding and noncoding sequences and generated amino acid changes, displaying functions not fully elucidated yet [52]. Furthermore, hyperedited *BLCAP* was associated with cervical cancer [53]. In this case, A-to-I RNA editing in the coding region of *BLCAP* altered the motif involved in the binding to the SH2 domain of the signal transducer and activator of transcription 3 (STAT3), with the subsequent loss of inhibition of STAT3 phosphorylation. Hence, editing of *BLCAP* could represent a driver event in the progression of cervical carcinogenesis [53].

In human hepatocellular carcinoma (HCC), ADAR1-editing activity on *AZIN1* transcript resulted in a serine-to-glycine substitution at residue 367 [54]. This causes a conformational change in the protein, leading to a cytoplasmic-to-nuclear translocation and a higher binding affinity to antizyme-1, resulting in increased AZIN1 stability [55]. The binding of AZIN1 inhibits antizyme-1-mediated binding and degradation of the ornithine decarboxylase (ODC) and cyclin D1 (CCND1) oncoproteins, contributing to cancer initiation and progression, and controlling the proliferative and invasive abilities of cells. In addition, in HCC samples, a higher editing level of *FLNB* (filamin B, β) and a lower editing level of *COPA* (coatomer protein complex subunit α) were found to be associated with pathogenesis [55]. A similar mechanism was found to drive the development of ESCC [56] and to regulate the malignant phenotype in NSCLC [57] and gastric cancer (GC) [58]. Moreover, in GC, the ADAR2 activity on the *PODXL* (podocalyxin-like) gene at codon 241, causing His-to-Arg substitution, indicated that *PODXL* is one of the ADAR2-editing targets responsible for its tumor-suppressive function [59]. In colorectal cancer (CRC), higher ADAR1 expression correlated with elevated RNA-editing levels of *AZIN1* [60], according to the mechanism described above. Furthermore, ADAR1 is overexpressed in fibroblasts from CRC specimens and conditioned medium derived from cancer cells and promotes *AZIN1* RNA editing in fibroblasts mediated by ADAR1 expression [61].

In addition, the RNA editing of the Ras homologue family member Q transcript (*RHOQ*) is higher in cancer tissue compared with normal tissue. As a result, the substitution of asparagine with serine at residue 136 (RhoQ N136S) increases RHOQ activity responsible for actin cytoskeleton reorganization and a higher invasion potential [62]. Another example of how RNA editing can act is given by the glioma-associated oncogene 1 (*GLI1*) factor, a key terminal effector of the hedgehog signaling pathway. The editing on the adenosine 2179 of *GLI1* mRNA leads to an amino acid change (from arginine to glycine at position 701) of the GLI1 protein that impacts its activity [63]. The increased Alu-dependent editing and transcriptional activity of GLI1 due to ADAR1 was observed also in multiple myeloma (MM) [63], where it promotes immunomodulatory drug resistance in vitro.

The A-to-I RNA-edited *GABRA3* was found only in noninvasive breast cancer. In fact, the edited form had reduced cell surface expression and suppressed the AKT-pathway activation required to promote cell invasion and metastasis [64].

The C-to-U editing in the *NF1* (neurofibromatosis type I) mRNA by APOBEC-1 creates an in-frame translation stop codon with a consequent reduction of its tumor-suppressor function [65]. Interestingly, in peripheral nerve-sheath tumor samples (PNSTs) from patients with NF1, C-to-U editing of NF1 was observed preferentially in an alternatively spliced form containing exon 23A [66].

In ER-positive breast cancer, the expression of the cytosine deaminase APOBEC3B (apolipoprotein B mRNA-editing enzyme, catalytic polypeptide-like 3B) inversely correlates with the response to tamoxifen treatment [67], suggesting a role for this enzyme in drug resistance. APOBEC3 protein was found to be involved in cancer development, due to its ability to affect the transcript of the tumor suppressor WT1 (Wilms Tumor 1). In fact, a noncanonical G-to-A RNA editing was found in the *WT1* transcript in nonprogenitor umbilical cord blood mononuclear cell samples (CBMCs), compared to acute myeloid leukemia (AML) [68].

Editing events discussed in the Section 3 were listed in Table 1.

### 3.1. Impact of RNA Editing on Splicing Decisions

**Figure 2:**
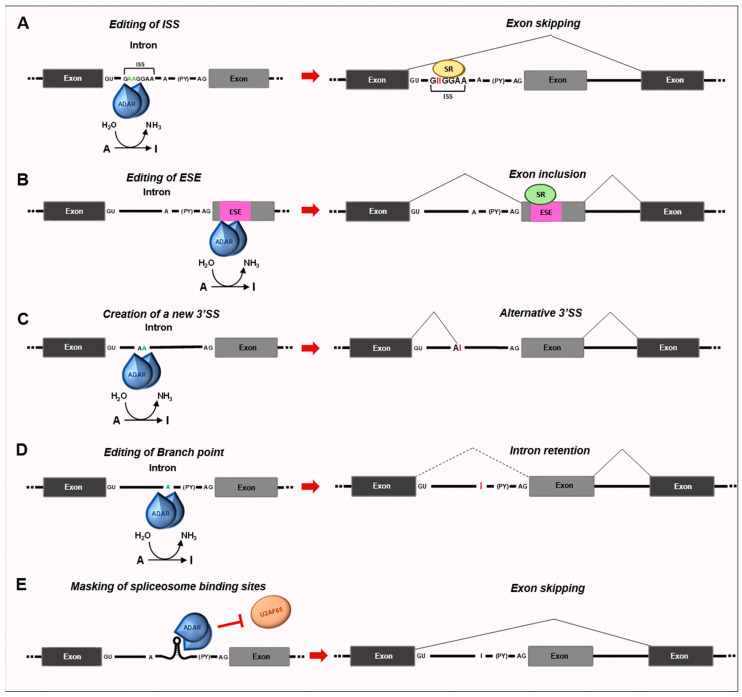
Schematic representation of the impact of RNA editing on the splicing outcome. (**A**,**B**) ADAR1 can bind and edit GA-rich motifs within *cis*-regulatory elements in introns and exons, thus affecting the binding of RNA-binding proteins. In particular, the A-to-I editing can empower the binding capacity of SR proteins, leading to exon skipping when the editing occurs in ISS (**A**), or exon inclusion when an ESE is edited (**B**). (**C**) Modification of AA sites within introns can result in AI mimicking AG and being recognized as new acceptor splice sites. (**D**) Deamination of the branch point adenosine can impair intron removal. (**E**) ADAR2 binding to dsRNA structures formed between GA-rich sequences located upstream of the poly-pyrimidine tract can sterically inhibit the access of U2AF65, thus preventing exon recognition. Abbreviations: SR, serine, and arginine-rich proteins; ISS, intronic splicing silencer; ESE, exonic splicing enhancer; Py, polypyrimidine tract; U2AF65, U2 small nuclear ribonucleoprotein auxiliary factor 65.

RNA editing extensively impacts alternative splicing choices. Editing can alter regulatory motifs, RNA–protein interactions, and RNA secondary structures, thus playing pivotal roles in modulating RNA processing (Figure 2). Several pieces of evidence demonstrated that editing occurs cotranscriptionally, prior to polyadenylation [69], which is corroborated by the fact that the C-terminal domain of RNA polymerase II (RNAPII) can facilitate site-specific editing [70]. In several genes, editing occurs prior to splicing. Accordingly, the reduction of ADAR expression is sufficient to induce global splicing changes [71]. Editing in introns can modify the consensus sequences contained in splicing enhancers and silencers, thus impairing the recruitment of splicing auxiliary proteins on the pre-mRNAs and, consequently, the splicing outcome [71]. Moreover, RNA editing can affect splicing by targeting the adenosines involved in the initial steps of spliceosome assembly. The conversion of intronic AA to adenosine–inosine (Al) dinucleotide mimics the highly conserved AG acceptor sequence at the 3′ splice junctions. This strategy is used by ADAR2 to modulate its own expression [72]. In rats, ADAR2 edits its own pre-mRNA to generate a functional 3′ splice site, leading to the recognition of a 47 bp exon which, when included, affects ADAR translation efficiency, by shifting the translation start to an inefficient downstream methionine [72]. Hence, the ability of ADAR2 to modulate alternative splicing may represent a negative autoregulatory mechanism to prevent its own overexpression to avoid aberrant editing. Notably, the inclusion of this exon was also detected in glioma cells, where the expression rate of this isoform was shown to correlate with malignancy [73]. Thus, the downregulation of A-to-I editing in gliomas might be due to the expression of a less-active isoform of ADAR2.

A-to-I editing of the *HNRNPLL* pre-mRNA by ADAR1 p110 and ADAR2 creates a binding site for SRSF1, leading to the inclusion of exon 12A and the consequent production of an hnRNPLL isoform displaying oncogenic activity in kidney and bladder tumors [74]. The creation or elimination of branch points by RNA editing can explain several editing-dependent splicing events. Transcriptome-wide RNA profiling identified 85 high-confidence splicing events regulated by ADAR1 and ADAR2 in esophageal squamous carcinoma (ESCC) cells [75], revealing the effect of editing on auxiliary *cis*-acting elements, which, in turn, impacts splicing factors binding affinity. 

ADAR proteins can influence splicing decisions by distinct mechanisms. Particularly, it was shown that ADAR1 binds to and edits an intronic silencer of *CCDC15*, located between introns 8 and 9, thus improving its strength and repressing exon 9 inclusion by enhancing the binding of the splicing repressor SRSF7 [75]. Conversely, ADAR2 can bind a GA-rich sequence near the polypyrimidine tract of the intron 2 of *RELL2*, therefore impeding the recognition of the 3′splice sites by U2AF65. As a consequence, exon 3 is skipped and causes a frameshift and premature stop codon, making the transcript susceptible to nonsense-mediated RNA decay (NMD) [75]. In this specific instance ADAR2 relies only on its ability to bind dsRNA to regulate and its catalytic activity is dispensable. Thus, ADARs can modulate exon choices through distinct mechanisms. The first one relies on the editing of GA-rich sequences within the intronic splicing silencer or enhancers, modulating sequence binding affinity for splicing factors. Alternatively, ADAR1 and ADAR2 can bind secondary structures formed by the pairing of the flanking introns of the exon cassettes, creating a loop that hampers the access to the spliceosome to the 5′ and 3′splice sites. Furthermore, ADAR2 can bind a GA-rich sequence near the polypyrimidine tract, therefore impeding the recognition of U2AF65 to the 3′splice sites [75]. These examples provide evidence that tumor-cell behavior is precisely regulated by the complementary function of ADARs and the crosstalk between editing and splicing machinery.

RNA-specific adenosine deamination of a branch site in *PTPN6* pre-mRNA was shown to give rise to a novel *PTPN6* transcript retaining intron 3, thus causing a nonsense translation of *PTPN6* mRNA and contributing to the production of a nonfunctional protein [76]. Notably, the level of the aberrant intron-retaining splice variant was lower in AML bone marrow mononuclear cells at remission than at diagnosis, suggesting the involvement of post-transcriptional *PTPN6* processing in leukemogenesis [76]. 

Human transcripts contain excess editing over mouse, rat, chicken, and fly transcripts [77], and more than 90% of known editing sites in humans are found in *Alu* elements, both in sense and antisense pairs of *Alus* [28]. *Alu* elements are unique primate-specific retrotransposons that occur in over one million copies of the human genome [77]. Exons containing *Alu* repeats, flanked by introns also encompassing *Alu*, are often targets of the editing machinery in their acceptor splice site, which is converted from AG to IG and interpreted as GG. The strength of the acceptor splice site is then dramatically reduced, causing the skipping of the exons [69]. Consistently, insertions of *Alu* elements within introns can result in the skipping of the adjacent exons. Insertion of an *Alu* element in the *NF1* caused skipping of the exon immediately downstream of the intron of insertion and, consequently, shifts in the reading frame, leading to neurofibromatosis [78]. 

Random mutations in *Alu* sequences can turn them into functional splice sites, recognized by the splicing machinery, thus contributing to the “exonization” process, even in a tissue-dependent fashion [79], as described for the *NARF Alu*-exon 8, “exonized” via tissue-dependent RNA editing [80]. In particular, the “exonization” of *Alu* elements in the *NARF* gene (encoding the nuclear prelamin A recognition factor), leads to the insertion of 46 in-frame additional amino acids in the encoded protein [80]. Similarly, A-to-I editing of *Alu* sequences in introns can also create a canonical *5*′ splice donor site GU (the result of AU-to-IU edit, recognized as GU by the splicing machinery) and/or a canonical *3*′ splice acceptor site AG (the result of an AA-to-AI edit, recognized as AG) which can influence the splicing outcome. This phenomenon has been observed in several transcripts, such as alternative splicing of exon 15a in the *GPR107* gene due to editing of the *AluSx* in intron 15 [25]. Editing of an intronic *Alu* sequence between exons 1 and 2 in the *SARS* gene (encoding seryl-tRNA synthetase) was also described to prevent aberrant “exonization” [81]. However, whether or not editing-induced “exonization” is involved in cancer transformation has not been elucidated yet.

Thus, these processing events deeply contribute to evolutionary transcriptomic processes, including the creation and deletion of exons [77]. In this way, *Alu* elements can increase the coding capacity of human genes while maintaining the original protein repertoire. However, to what extent this process affects transformation has not been deeply elucidated yet. Recently, it was shown that editing of the *PODXL* pre-mRNA promotes the inclusion of an alternative exon [82]. The resulting edited *PODXL* long isoform is more prone to protease digestion and reducing cell migration and cisplatin chemoresistance [82]. Remarkably, the editing level of the *PODXL* transcript and the inclusion level of the *PODXL* alternative exon were strongly associated with overall patient survival in kidney renal clear cell carcinoma (KIRC) [82]. 

Overall, the creation or elimination of splice sites and branch points by RNA editing can explain several editing-dependent splicing events [70,72,76,80]. Hence, RNA editing promotes proteomic diversity not only through amino acid changes but also through alternative splicing, recoding cancer cells, and contributing to therapeutic resistance.

Editing events affecting splicing decisions were listed in Table 2.

### 3.2. Impact of RNA Editing on miRNA Function

**Figure 3:**
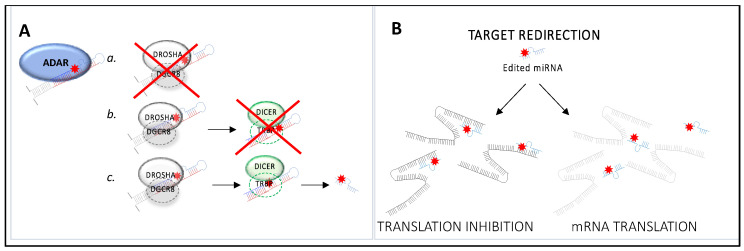
RNA editing can affect miRNA biogenesis. (**A**) In the nucleus, the RNase III Drosha is the core nuclease that executes the initiation step of miRNA processing. MicroRNA molecules are synthesized as long RNA primary transcripts known as pri-miRNAs, which are cleaved by Drosha to produce a characteristic stem-loop structure of about 70 base pairs long, known as a pre-miRNA. DGCR8 is essential for Drosha activity and is capable of binding single-stranded fragments of the pri-miRNA that are required for proper processing. In the cytoplasm, pre-miRNA is further processed into mature miRNA by the RNase Dicer, in complex with TRBP. A-to-I RNA editing can prevent the processing of pri- (**a**) or pre-miRNA (**b**). In certain cases, the A-to-I editing event does not impair miRNA biogenesis, and the edited nucleotide is maintained in the mature miRNA (**c**), determining target redirection (**B**).

Editing of miRNA precursors displays significant implications on miRNA fate and function, affecting both the expression of the miRNA and its ability to recognize target sequences (Figure 3 and Table 3). A-to-I editing of miRNA precursors can alter their primary structure, as reported for over 550 human miRNA transcripts [83], and lead to structural conformation changes which can prevent Drosha or Dicer from processing them (Figure 3A). MiRNA molecules are synthesized as long RNA primary transcripts known as pri-miRNAs, which are then cleaved by Drosha to produce a characteristic stem-loop structure of about 70 base pairs long, known as a pre-miRNA. DGCR8 is essential for Drosha activity and is capable of binding single-stranded fragments of the pri-miRNA that are required for proper processing (Figure 3A) [84]. Pre-miRNAs are further processed into mature miRNAs by the RNase dicer (Figure 3A) [84]. A-to-I RNA editing can prevent the processing of pri- or pre-miRNAs. However, in certain cases, the A-to-I editing event does not impair miRNA biogenesis, and the edited nucleotide is maintained in the mature miRNA. Since miRNA-mediated translational repression is conferred by sequence complementarity between miRNA seed regions and seed-complementary sequences within the target mRNA, even a single nucleotide substitution along the mature miRNA sequence can determine changes in its target repertoires, causing target redirection, in which novel miRNA targets are created and/or complementarities between miRNAs and the UTRs of canonical targets are destroyed [85] (Figure 3B).

Recently, the aggressiveness of thyroid cancer has been associated with the overedited miRNA200b by ADAR1 [86]. The 3′ UTR of the epithelial–mesenchymal transition (EMT) marker ZEB1 is the target of miRNA200b, with a consequent decrease in its expression. Overediting impairs the ability of miRNA200b to inhibit ZEB1, inducing an alteration of cancer-cell aggressiveness. RNA editing of miR-455-5p by ADAR1 determines its ability to recognize a different set of genes in melanoma cells [87]. In particular, the edited miR-455-5p specifically targets the tumor-suppressor gene CPEB1, reducing melanoma growth and metastasis. In a similar manner, the regulation of miR-376a editing was found relevant in human glioma [88]. The unedited miR-376a promoted glioma cell migration and invasion through its ability to target the 3′UTR of RAP2A and its inability to target AMFR (autocrine motility factor receptor) due to a single base difference (in the edited form). Moreover, in melanoma cells, ADAR1 activity on the miRNA-378a-3p enhances its binding to the 3′-UTR of the PARVA oncogene, inhibiting its expression and preventing melanoma progression [89]. In chronic lymphocytic leukemia (CLL), the overexpression of the *ADARB1* gene is associated with reduced processing of miRNA-15/-16, suggesting a novel oncogenic mechanism in CLL [90]. In addition, Gassner and colleagues provide evidence that miR-3157 and miR-6503 editing occurs specifically in CLL, and this editing pattern is associated with a shorter progression-free survival (PFS) in patients [91]. Furthermore, an impaired editing function renders CLL cells more susceptible to different therapeutic regimens in vitro [92]. Importantly, miRNA editing was also found in circulation. By analyzing small-RNA sequencing data from exosome samples from NSCLC patients at different stages, miR-411 edited in position five was found differentially expressed between NSCLC and normal tissue samples [93].

A-to-I editing can induce changes in the specific miRNA target sequences, as observed for the transcript encoding the Rho GTPase activating protein 26 (*ARHGAP26*) [94]. In cancer specimens, ARHGAP26 expression was found to be downregulated due to its targeting by miR-30b-3p and miR-573. Interestingly, in human invasive ductal breast cancer and glioblastoma, the putative binding sites of miR-30b-3p and miR-573 in the 3′ UTR of *ARHGAP26* transcript are destroyed by the ADAR1 editing activity leading to increased *ARHGAP26* expression, cell spreading, and migration [94]. Overall, edited miRNAs might represent efficient diagnostic and prognostic biomarkers of cancer development and progression, exploitable as novel therapeutic targets.

**Table 3:** List of miRNAs whose editing has a role in cancer.

Type of Cancer	Enzyme Involved	Type of Activity	miRNA Name	Downstream Effect
**Thyroid cancer** **[86]**	ADAR1	Overedited (A-I)	***miR-200b***	Alteration of miRNA ability to target 3′UTR of *ZEB1*, promoting cancer aggressiveness
**Melanoma** **[87]**	ADAR1	Underedited (A-I)	***miR-455-5p***	Binding to the 3′UTR of *CPEB1*, promoting cancer progression
**[89]**	ADAR1	Overedited (A-I)	***miR-378a-3p***	Binding to the 3′UTR of *PARVA*, preventing cancer progression
**Chronic lymphocytic leukemia** **[90]**	ADARB1	Overedited	***miR-15/16***	Dysregulated miRNA processing, promoting cancer progression
**[91]**	ADAR1	Overedited	***miR-3157*** ***miR-6503***	Shortened Progression-Free Survival
**Nonsmall cell** **lung cancer** **[93]**	ADAR	Underedited (A-I)	***miR-411-5p***	Changes in miRNA targets, promoting cancer
**Breast cancer** **[94]**	ADAR1	Overedited (A-I)	***miR-30b-3p*** ***miR-573***	Alteration of miRNA’s ability to target 3′UTR of *ARHGAP26*, promoting cancer progression
**Glioblastoma** **[88]**	ADARB1	Underedited (A-I)	***miR-376a***	Binding to the 3′UTR of *RAP2A*, promoting invasiveness;
**[94]**	ADAR1	Overedited (A-I)	***miR-30b-3p*** ***miR-573***	Alteration of miRNAs ability to target 3′UTR of *ARHGAP26*, promoting cancer progression

## 4. ADAR RNA Editing as a Novel Biomarker for Cancer Diagnosis and Treatment

As discussed above, the most studied mechanism of RNA editing in cancer is the deamination of adenosine due to the ADAR family members. Several studies have highlighted abnormal RNA-editing levels and the aberrant expression of the editing enzymes in cancer specimens compared to normal tissues, indicating that aberrant editing could play a role in cancer development and progression. However, A-to-I editing levels correlate with an enhanced or reduced tumoral phenotype depending on the specific cancer type [95,96]. Although, in some cases, RNA-editing events may act as “drivers” for tumor growth and serve as prognostic or predictive markers for patient stratification, just like somatic mutations, in other cases, the correlation is not clear. Thus, the evaluation of the prognostic impact and target modality of RNA editing in therapeutic approaches is very challenging and controversial. Nevertheless, establishing an accurate diagnosis in early tumor stages might improve patients’ clinical prognoses, clarifying the processes underlying the evolution. Treatments should be formulated after an accurate analysis of the editing pattern in cancer-related genes and on the stratification of tumors based on the underedited or overedited cancer type [95,97]. The combination of machine-learning methods and bioinformatics may provide more innovative solutions in personalized medicine. In this regard, based on a comprehensive analysis of the general pattern of RNA editing in patients with low-grade glioma, Wang and colleagues were able to construct a model consisting of four RNA-editing sites as predictors of patients’ survival, confirmed by machine-learning algorithms [98]. This tool might help optimize survival risk assessment and individualized care. Another relevant factor is represented by the contribution of the different ADAR enzymes on the specific cancer-related editing profile. In breast cancer, a globally higher A-to-I editing frequency was described, in comparison to normal tissues, mostly accountable to the ADAR1, but not to ADAR2, activity [43,99]. Furthermore, the recoding activity and the expression of ADAR1, but not ADAR2, correlates with the overediting of specific oncogenic or tumor-suppressor transcripts in different tumor types [53,54,56]. Remarkably, even if ADAR1 and ADAR2 can target the same transcript [52], their functions are not redundant or compensatory [100], as suggested by the lethality of mice knockout for either ADAR1 or ADAR2 [101,102]. These findings underline the possibility of developing a selective therapy to target ADAR1 without the offsetting of ADAR2. With this aim, it was designed as an adenosine analog (8-azaadenosine), capable of selectively inhibiting ADAR1 activity. This compound was able to attenuate the malignant phenotype of thyroid cancer in vitro [86] but triggered adverse effects when administered in rat models [103]. These contradictory data denoted the necessity of developing other molecules or different approaches, more tolerated by living organisms, to reduce ADAR1 activity in cancer. 

In mice, ADAR1 deletion has been associated with a loss of leukemic cells and a reduction of the leukemic cell burden in the spleen [104]. In addition, three recent works based on CRISPR-Cas9-mediated loss of ADAR1 function in different cancer types showed that ADAR1 deletion reduced cell viability and increased the sensitivity to immunotherapy. The mechanism relies on the recovered activation of the IFN pathway, which inversely correlates with hyperediting reduction [105,106,107]. This approach was not successful because it did not specifically target cancer cells, and ADAR1 deletion in normal cells is not compatible with life. Notably, in several malignancies it was described as a remarkable decrease in ADAR2 activity, correlating with tumor aggressiveness [44,49,52,108]. This could be due to an antagonistic effect of ADAR1 that sequesters ADAR2 by forming inactive heterodimers [44]. In fact, ADAR1 exists as two different isoforms, a short one named p110 and constitutively expressed in the nucleus, and a longer form named p150, localizing both in the nucleus and the cytoplasm and interferon inducible [109]. Since ADAR1 p110 is overexpressed in different tumors [44,107] and is responsible for the sequestration of ADAR2 [44], a possible approach to rescue ADAR2 activity in cancer could be to design specific small molecules or peptides inhibiting the interaction between ADAR1 p110 and ADAR2. Given the high homology of ADAR enzymes and the activity of ADAR2 linked to its homodimerization [110], this strategy might be tricky and not selective. A very interesting and recent discovery identified the DEAH box helicase 9 DHX9 as a bidirectional regulator of ADAR1 and ADAR2 activity in different cancers [111]. Notably, DHX9 is upregulated in several tumors [112,113,114,115], and it has been found to exert an oncogenic role by eliciting ADAR1 editing and contemporary repressing ADAR2 activity [111]. As mentioned above, ADAR1 and ADAR2 show a preference for editing sites with precise adjacent nucleotides, but no conserved motifs have been determined, suggesting that the preference may be due to the RNA’s local structure. Thus, the opposite regulatory effect of DHX9 may be in part due to its helicase activity, but the two different structural signatures have not been deciphered yet. In the same context, DHX9 has been found to interact with ADAR1 p110 and ADAR2 in the nucleus [111], but the relevance of this dual interaction in the regulation of RNA editing has not been deeply investigated. Possibly then, the design of specific peptides or small molecules capable of selectively inhibiting the interaction between DHX9 and ADAR1 could be a successful trick for repressing ADAR1 oncogenic hyper-editing and recovering ADAR2 activity, to weaken tumor phenotype. 

Another interesting aspect to consider for the treatment of cancer is the connection between RNA editing and splicing [116,117,118]. As mentioned above, A-to-I editing of hnRNPLL by ADAR1 p110 and ADAR2 creates a binding site for SRSF1, leading to the inclusion of exon 12A and the consequent production of an hnRNPLL protein isoform with oncogenic activity in kidney and bladder tumors [74]. In another study, ADAR1 and ADAR2 were shown to repress exon inclusion in two different transcripts (*CCDC15* and *RELL2*), thereby leading to the production of pro-oncogenic protein isoforms [75]. Interestingly, this study revealed that ADARs can modulate exon inclusion through distinct mechanisms. The first one relies on the editing of a GA-rich sequence within an intronic splicing silencer by ADAR1 and ADAR2 and the subsequent creation of a binding site for SRSF7, leading to the repression of exon inclusion. ADAR1 and ADAR2 can also bind the dsRNA structures formed by the pairing of the flanking introns of the exon cassette, creating a loop that denies access to the spliceosome to the 5′ and 3′SS. Alternatively, ADAR2 can bind a GA-rich sequence near the polypyrimidine tract, thereby impeding the recognition of U2AF65 to the 3′SS. These results suggest that there exists in cancer a fine-tuned regulation of RNA editing, and, even if the preponderance of editing events in human cells befalls in repetitive element and noncoding sequences [119], the study of the edited sites may unravel new splicing products. An aberrant splicing pattern, then, may be targeted with splice-switching antisense oligonucleotides (ASOs), inducing inclusion/skipping of selected exons by annealing to the complementary sequence of a target pre-mRNA and, thus, blocking the binding of splicing factors [120]. ASOs are efficient therapeutic tools for spinal muscular atrophy [121], that may be conceivably useful for cancer treatments. Interestingly, ADAR2 can autoedit itself, thereby affecting its alternative splicing. In fact, A-to-I editing within intron 1 mimics a 3′SS and causes a 47-nt insertion and the production of an alternative splice variant with decreased catalytic activity [72]. Notably, the ADAR2 transcript has nine alternative splicing sites that produce different protein isoforms with increased or decreased activity [122], and the expression of the variant including exon 5A, which losses 50% of its activity, has been correlated with the underediting of the GluR-B Q/R site and the glioma pathogenesis [123]. Hence, it is reasonable to believe that the analysis of the ADAR2 splice variants in tumors with decreased ADAR2 activity might be useful to understand whether the ASOs treatment against ADAR2 could restore a normal editing profile, weakening the malignant phenotype.

## 5. Conclusions

RNA editing opens a novel layer of complexity to the intricate process underlying cancer development, progression, and resistance to therapy. RNA-editing events occurring in tumors have allowed for the identification of the pathological mechanisms specifically linked to the different stages of cancer progression. Understanding these signatures could contribute to the development of more efficient patient-tailored anticancer therapies. RNA-editing events could even be used diagnostically as early biomarkers for ongoing tumor diversification and relapse. On the other hand, pharmacological modulation of writers, readers, and erasers by specific inhibitors or activators could have huge therapeutic potential. In this study, we have reviewed editing changes occurring in cancer; some of them represent only an accompanying phenomenon, not necessarily a contributing factor to cancer progression, while others may play a direct role in cancer development and affect therapeutics. Therefore, some RNA-editing changes might be considered as cancer biomarkers but not therapeutic targets. To date, more effective strategies to identify, validate, and analyze editing events are still needed to allow for precision medicine based on epi-transcriptome signatures that are tailored to specific tumor types. Hence, more efforts should be made in the coming years to characterize editing in normal and cancer conditions, providing the opportunity to develop more sophisticated diagnostic and prognostic procedures in clinical practice.

## Figures and Tables

**Table 1:** List of editing events that affect transcript stability or produce changes in the amino acid sequence of the translated protein.

Type of Cancer	Enzyme Involved	Type of Activity	Downstream Effect
**Nonsmall-cell lung cancers** **[46]**	ADAR	Overedited (A-I)	Stabilization and increase of *FAK* transcript, promoting tumor progression
**[57]**	ADAR	Overedited (A-I)	Alteration of the *AZIN1* transcript, resulting in nuclear translocation, promoting the malignant phenotype
**Esophageal Squamous Cell** **[47]**	ADAR2	Overedited (A-I)	Decrease of *SLC22A3* transcript, promoting tumor progression
**[56]**	ADAR1	Overedited (A-I)	Alteration of the *AZIN1* transcript, promoting tumor progression
**Breast Cancer** **[48]**	ADAR1	Overedited (A-I)	Alteration in *GINS4* transcript stability, alteration in *ATM* and *POLH* transcript expression, promoting tumorigenesis
**[64]**	ADAR1	Overedited (A-I)	Alteration of the *GABRA* transcript, causing amino acid substitution and suppressing tumor progression
**[67]**	APOBEC3B	Underedited (C-U)	Not investigated
**Astrocytoma** **[49]** **[44]**	ADAR2 ADAR1/2	Underedited (A-I) Underedited (A-I)	Alteration of the *CDC14B* pre-mRNA, increasing its expression with consequent reduction of tumorigenicity Not investigated
**Glyoblastoma** **[51]**	ADAR2	Underedited (A-I)	Alteration of the *GluR-B* transcript, causing amino acid substitution and tumor invasiveness
**Pediatric Astrocytoma** **[44]**	ADAR2	Underedited (A-I)	Not investigated
**Cervical Cancer** **[53]**	ADAR1	Overedited (A-I)	Alteration of the binding motif of *BLCAP* for STAT3, promoting tumor progression
**Hepatocellular carcinoma** **[54]**	ADAR1	Overedited (A-I)	Alteration of the *AZIN1* transcript, causing amino acid substitution and promoting tumor progression
**Gastric Cancer** **[58]**	ADAR1	Overedited (A-I)	Not investigated
**[59]**	ADAR2	Overedited (A-I)	Alteration of the *PODXL* transcript, causing an amino acid substitution, reducing tumorigenicity
**Colorectal cancer** **[60]**	ADAR1	Overedited (A-I)	Alteration of the *AZIN1* transcript, promoting ODC accumulation and tumor progression
**[62]**	ADAR	Overedited (A-I)	Alteration of the *RHOQ* transcript, causing an amino acid substitution and promoting tumor progression
**Multiple Myeloma** **[63]**	ADAR1	Overedited (A-I)	Alteration of the *GLI1* transcript, causing an amino acid substitution and promoting tumor progression
**[68]**	APOBEC3A	Overedited (G-A)	Alteration in *WT1* transcript, promoting tumorigenesis

**Table 2:** List of editing events affecting the splicing process.

Type of Cancer	Enzyme Involved	Type of Activity	Gene Name	Downstream Effect
**Kidney and bladder [74]**	ADAR1/2	Overedited (A-I)	***HNRNPLL***	Creation of a binding site for SRSF1 and inclusion of exon 12A, promoting tumorigenesis
**Esophageal squamous carcinoma** **[75]**	ADAR1/2	Overedited (A-I)	***CCDC15***	Enhancing the binding sites for SRSF7 and repression of exon 9 inclusion, promoting tumorigenesis
**Acute myeloid leukemia** **[76]**	Not reported	Overedited (A-I)	***PTPN6***	Retaining of intron 3, causing a nonsense translation, favoriting leukemogenesis
**Glyoblastoma** **[81]**	ADAR1	Overedited (A-I)	***SARS***	Preventing aberrant exonization of *Alu* sequence into mature mRNA, protecting by degrading aberrant transcripts
**Kidney Renal Clear Cell Carcinoma** **[82]**	ADAR2	Underedited (A-I)	***PODXL***	Inclusion of an alternative exon and production of a longer isoform, promoting cell migration

## Data Availability

The data can be shared up on request.
